# Initial Experience with LVIS EVO Stents for the Treatment of Intracranial Aneurysms

**DOI:** 10.3390/jcm9123966

**Published:** 2020-12-07

**Authors:** Wojciech Poncyljusz, Kinga Kubiak

**Affiliations:** Department of Diagnostic Imaging and Interventional Radiology, Pomeranian Medical University, 71-252 Szczecin, Poland; wojciech.poncyljusz@pum.edu.pl

**Keywords:** aneurysm, LVIS EVO, embolization

## Abstract

Background: Over the years, a variety of intracranial stents have been developed, which has expanded the therapy options available for cerebral aneurysms. The Low profile visible intraluminal support (LVIS) EVO stents are new devices, which officially appeared on the market in 2020. The purpose of the study is to report the initial technical and clinical experience with the new stent in the treatment of intracranial aneurysms. Materials and Methods: Between February and September 2020, 30 patients with 35 intracranial aneurysms (29 unruptured and 6 ruptured) were treated using the LVIS EVO stent in our department. The aneurysms were located within internal carotid artery (ICA) (42.9%), middle cerebral artery (MCA) (31.4%), anterior communicating artery (AComA) (11.4%), basilar artery (BA) (11.4%) and anterior cerebral artery (ACA) (2.9%). Stent-assisted coil embolization was performed in all cases. Results: All stents were deployed successfully in the desired position. Immediate complete occlusion of the treated aneurysms, described as Raymond–Roy occlusion classification (RROC) class 1, was achieved in all cases. No technical complications were observed. One thromboembolic complication occurred in the group of unruptured aneurysms and one patient died due to cerebral edema from aneurysms rupture group. Conclusion: In our observation, the showed a satisfactory safety profile LVIS EVO stents seem to be very flexible, can be safely maneuvered and deployed in tortuous vessels. They showed a good initial occlusion rate when used for treating intracranial aneurysms with SAC (stent-assisted coiling).

## 1. Introduction

Endovascular treatment of unruptured and ruptured intracranial aneurysms has become a favorable option in the majority of cases, in comparison to surgical clipping [1,2,3,4]. Stent-assisted coiling (SAC) enables endovascular management of complex and wide-necked aneurysms with low procedure-related morbidity and mortality [5,6,7,8]. Stents provide several benefits, e.g., they prevent the protrusion or dislocation of coils into the parent artery, promote vessel wall healing, and some reduce intra-aneurysmal flow due to a partial flow-divert effect [9,10].

LVIS EVO (MicroVention-Terumo, Aliso-Viejo, CA, USA) is one of the most recent innovations, and it is a self-expandable stent designed for use in cerebral vasculature. This stent consists of 16 nitinol wires with a platinum core, and its wires are braided using the DFT (drawn filled tube) technology, which makes all the wires visible in fluoroscopy. It makes the stent easy to manipulate in tortuous vessel anatomy and provide enhanced opening ability.

These stents are characterized by very short flared ends (0.5 mm), which provides adequate anchorage and makes the landing zone planning more flexible. Each end of the stent has four radiopaque markers. The stents are available in five different diameters (2 mm–4 mm), their length varies from 12 to 34 mm. The potential differences in comparison to other braided stents, such as LVIS Jr. (MicroVention-Terumo, Aliso-Viejo, CA, USA), include shorter flared ends, smaller cell size and enhanced visibility.

The paper reports the initial technical and clinical experience with the use of the LVIS EVO stent in the treatment of ruptured and unruptured cerebral aneurysms.

## 2. Materials and Methods

It was a single-center study, in which clinical and radiological data were collected prospectively and then retrospectively reviewed. Clinical history, aneurysm location and morphology, technical difficulties, and complications were analyzed. The grade of occlusion was evaluated using the Raymond–Roy occlusion classification (RROC) scale. The study protocol was approved by the Bioethics Committee no. KB-0012/29/05/2020/Z. Written informed consent was obtained prior to the treatment from all the patients. Severely affected patients with acute intracranial hemorrhage due to aneurysm rupture were treated as an emergency indication. All endovascular procedures were performed on Philips (Azurion Clarity IQ-Medical Systems Nederland BV), under general anesthesia, by the same interventional neuroradiologist. All patients with planed unruptured aneurysms were premedicated with antiplatelet therapy consisting of 150 mg of acetylsalicylic acid and 75 mg of clopidogrel daily for 7 days before the procedure. Patients with ruptured aneurysms received a loading dose of 600 mg clopidogrel and 300 mg of acetylsalicylic acid 2 h before the treatment. Dual antiplatelet therapy (acetylsalicylic acid 150 mg, clopidogrel 75 mg) was continued for 3 months post-operatively.

**Figure 1:**
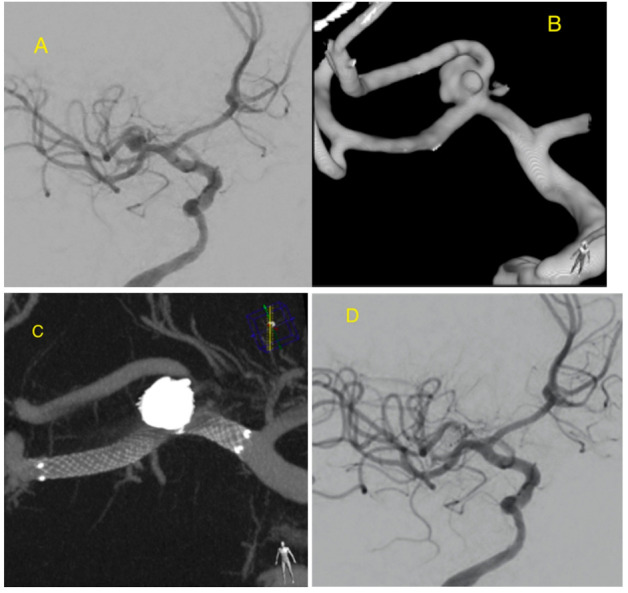
Right MCA bifurcation aneurysm visible on digital subtraction angiography (DSA) (**A**) and 3D reconstruction of a rotational angiography (**B**) demonstrates the aneurysm morphology of the sac and neck. The aneurysm was stented and coiled using jail technique. Excellent opposition of the stent to the vessel wall. Visible support of the coils with a stent arm which protects the aneurysm neck and parent artery (**C**). DSA shows aneurysm occlusion (**D**).

**Figure 2:**
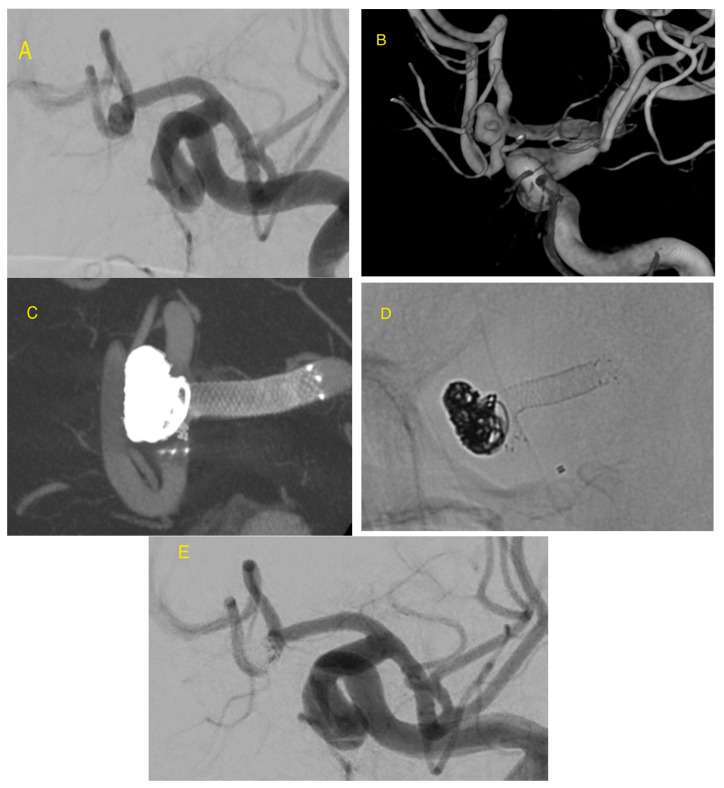
AComA wide-necked aneurysm is seen on DSA (**A**). Three-dimensional reconstruction of a rotational angiography (**B**) demonstrates the complex aneurysm morphology. LVIS EVO stent assisted coiling was used to treat this ruptured aneurysm. Flat panel detector computed tomography (CT) and Maximum Intensity Projection (MIP) reconstruction (**C**) and X-ray picture (**D**) showing very flexible stent arm, angled 90 degrees, protects completely the wide neck of the aneurysm from coil protrusion. DSA shows aneurysm occlusion (**E**).

Endovascular access, using Seldinger technique, was established via the common femoral artery using a 6F sheath introducer (Cordis, Bridgewater, NJ, USA). A bolus of heparin was administered via the sheath. Heparin was contained in the flush systems used for microcatheters (1000U per liter in unruptured and 500U in ruptured aneurysms). The guiding catheter Chaperon 6F (MicroVention-Terumo, Aliso-Viejo, CA, USA) was positioned in the C1 segment of the internal carotid artery (ICA) or V1 segment of the vertebral artery (VA) depending on the location of the aneurysm. Cerebral DSA (digital subtraction angiography) and then rotational angiography with 3D reconstruction were performed before treatment in order to determine the aneurysm morphology, local vascular anatomy, and the 3D roadmap to maneuvering neurointerventional equipment. Precise measurement of the diameter of the proximal and distal vessel to the aneurysm allowed us to select a suitable stent. The Traxcess 0.014-inch microguidewire (MicroVention-Terumo, Aliso-Viejo, CA, USA) and Headway 17 microcatheter (MicroVention-Terumo, Aliso-Viejo, CA, USA) were used under 3D roadmap to obtain the correct position for stent implantation. In all cases, embolization was performed using detachable coil systems: 3D or 360 Cosmos as the first coil, then Helical Soft or HyperSoft (MicroVention-Terumo, Aliso-Viejo, CA, USA) after stent deployment. Direct visualization in fluoroscopy with a 3D roadmap during partial deployment confirmed the position of the stent. If reposition was required, the stent was resheathed easily and redeployed in the adequate position. The push and pull technique was used to deployed stent on cured vessels to accurately match the stent surface to the artery wall. After stent implantation and coiling, DSA and flat-panel computed tomography (Vaso-CT) with reconstruction (Figure 1 and Figure 2) were performed to evaluate the final position of the stent. The degree of aneurysm occlusion was evaluated using the Raymond–Roy occlusion classification (RROC) afterwards. The femoral artery puncture site was closed with the vascular closure system—FemoSeal (70%) or AngioSeal (Terumo Europe) (26.7%). In one case (3.3%), manual compression was applied. Clinical evaluation was performed using the modified Rankin Scale (mRS) by a neurosurgeon before the procedure, after embolization, and at discharge.

## 3. Results

In total, 30 patients with 35 aneurysms were treated with the LVIS EVO stent-assisted coiling between February and September 2020. The patients’ characteristics are summarized in Table 1. The mean age was 60.7 years (range 4–78). Twenty-four were females and six were males. The aneurysms were located on the internal carotid artery 15 (42.9%), the middle cerebral artery 11 (31.4%), anterior communicating artery 4 (11,4%), basilar artery 4 (11.4%) and the anterior cerebral artery 1 (2.9%).

The mean aneurysm size was 7.11 mm (range 1.8 mm–21.66 mm). The average neck size was 4.8 mm (range 1.95–10.06 mm). Sixteen aneurysms were wide-necked (neck > 4 mm). The mean proximal diameter of parent artery was 3.01 mm and mean distal diameter was 2.57 mm.

The majority of aneurysms (71.4%) were incidental, with history of headache as the reason for clinical consultation. Seven patients had multiple aneurysms. The aneurysms were located close enough for single stent in three patients, providing support for the coil embolization. Four patients had undergone endovascular coil embolization prior to the procedure, and follow-up angio-MR showed dome or neck remnants, requiring re-interventions. Six patients presented with subarachnoid hemorrhage (SAH) related to aneurysm rupture. One patient with a ruptured ICA aneurysm died due to cerebral edema (Hunt/Hess grade 4). Characteristics of patients with ruptured aneurysms with SAH are summarized in Table 2.

One procedure-related complication occurred. The controlled angiography showed a distal thromboembolic event, most likely due to a formation of a small clot at the distal part of the guiding catheter which was broken off from the end of the system. The most likely cause of this was folding of the guide catheter and to slowing flow of saline in it. It was successfully treated with thrombectomy. There were no stent-related complications. All stents were delivered and accurately positioned in the desired location and position. All of the aneurysms were treated with detachable coils also. The postprocedural DSA demonstrated an excellent result with no residual filling of aneurysm and good perfusion beyond the stent (RROC I) in all cases.

## 4. Discussion

According to the International Study of Unruptured Intracranial Aneurysms (ISUIA) and the International Subarachnoid Aneurysm Trial (ISAT), endovascular coil embolization of unruptured and ruptured cerebral aneurysms is associated with a lower immediate morbidity and mortality in comparison to surgical clipping [3,4]. However, the treatment of wide-necked (>4 mm or dome-to-neck ratio <2) and giant (>25 mm) aneurysms presents higher rates of incomplete occlusion and remains technically challenging [5,6,7,8]. The development of various stent systems has greatly extended the applicability of endovascular treatment [11]. Stent-assisted coiling (SAC) improves safety in treatment of wide-necked and complex aneurysms, as it prevents coil herniation or migration into the lumen of parent artery. Such stents provide mechanical support as well as a scaffold for endothelialization, which facilitates aneurysm thrombosis [12,13]. Nowadays, a number of various stents, with different design and configuration, are available on the market. Braided or laser cut stents, which can be classified as open-cell or closed-cell depending on the struts’ structure, have different advantages and disadvantages [6,14,15]. Braided stents, which are designed for stent-assisted coiling, exhibit flow diverting properties [16]. As compared to open-cell stents, they exhibit less disadvantageous behavior and can be used in a wider range of complicated cases [14,17]. Laser-cut closed-cell stents may develop kinks in arterial bends. The lack of re-sheathing constitutes a significant disadvantage of those stents, as this feature is necessary in cases of high vessel tortuosity, and its lack can cause dislocation during deployment [18]. 

Few studies comparing in-vitro behaviors of intracerebral stents have been published. According to Cho et al. [19] Leo and LVIS stents, in comparison to Neuroform and Enterprise, have the highest radial force. In a study by Li et al. [20], comparing a braided and laser-cut stent (the LVIS and Enterprise), showed that there is no significant difference regarding periprocedural complications and immediate and mid-term follow-up, although the LVIS stents are characterized by lower recanalization rate and in-stent stenosis.

The LVIS EVO device is a self-expanding, closed-cell stent, with substantial differences to the LVIS and LVIS jr. stent (Low-profile Visualized Intraluminal Support). The LVIS EVO has DFT, which makes it easy to visualize and this, in turn, facilitates stent deployment, which is similar to different Leo stents (Balt, Montmorence, France) [14,21] and Accero (Accandis, Pforzheim, Germany) [22]. Another advantage of DFT is the influence on geometry, formability and opening ability of the stents. Based on the results of this study, the LVIS EVO seems to be very flexible, in a similar manner that flow diverters are, and is well adapted to the vascular anatomy. In our study, all stents were deployed in the desired location without any procedure-related complications; however, a shortening of the proximal part should be taken into consideration during implantation. The low profile of delivery microcatheters constitutes another advantage of this stent, as they can be navigated to distally located aneurysms more easily. Our series included 16 aneurysms that were located within the anterior communicating, middle or anterior cerebral artery, and were treated using LVIS EVO without any procedural difficulties in reaching and stenting the target vessel. The LVIS EVO stent has a high metal coverage of up to 28%, depending on the size of the implanted stent and the diameter of the parent vessel, and this value is significantly higher than for laser-cut stents and other braided stents [19]. This stent can generate the flow diverting effect, as the maximum metal coverage is as high as the minimum metal coverage of Flow Re-Direction Endoluminal Device (FRED) Jr. flow divert, whose range is between 28 and 33% [23]. Probably except coiling, the additional flow divert stent effect may explain that immediate control angiography showed complete occlusion in all aneurysm cases in our study. The LVIS EVO stents are characterized by small cell size, which can be disadvantageous, as the size may hinder passing through the stent mesh with microcatheter for coiling. The jailing technique was recommended and used in our group of patients as first-line technique.

There is only one publication demonstrating technical success of LVIS EVO [24]. In this paper, a group of six patients showed complete occlusion of all the aneurysms with no intraprocedural complications. Our initial experience with LVIS EVO stents is very positive too. Due to the enormous adaptability to the aneurysm neck even in cases with very difficult and tortuous anatomy with broad neck (using the push and pull technique), this stent is very effective and appears to be an alternative to treat cases qualified for such devices as WEB, P-Conus or PulseRider [25,26,27]. In our study, one distal thromboembolic complication that was not stent-related occurred, treated with thrombectomy, and no permanent neurological deficit remained post-operatively. There are some limitations to our study. The LVIS EVO stent was only recently introduced to the market, and this is the reason we are not able to present the results after a 6-month follow-up period in the majority of cases yet. We do not have Cor-lab evaluation for our cases. However, a favorable result over a longer term may be expected, as the rate of immediate occlusion was high. Nevertheless, a long-term follow-up is essential, and the patients remain under constant supervision.

## 5. Conclusions

The LVIS EVO is a new generation stent that ensures high safety and efficacy in the treatment of brain aneurysms. Long-term follow-up is essential.

## Figures and Tables

**Table 1:** Patients’ characteristics.

Mean Age (years)	60.76	
Gender (female/male)	24 (80%)/6 (20%)	
Aneurysm Characteristics		
Aneurysm location	ICA	15 (42,9%)
	MCA	11 (31.4%)
	AComA	4 (11.4%)
	BA	4 (11.4%)
	ACA	1 (2.9%)
Aneurysm Status		
Incidental	25	
Recanalized	4	
Ruptured	6	
Aneurysm Size		
<7 mm	20	
>7 mm	15	
Aneurysm Neck		
<4 mm	19	
>4 mm	16	

Internal carotid artery (ICA), middle cerebral artery (MCA), anterior communicating artery (AComA), basilar artery (BA) and anterior cerebral artery (ACA).

**Table 2:** Characteristics of patients with ruptured aneurysms.

**Hunt–Hess Scale**	
Grade 1	2 patients
Grade 2	1 patient
Grade 3	1 patient
Grade 4	2 patients
**Fisher Scale**	
Grade 2	3 patients
Grade 3	1 patient
Grade 4	2 patients

## Abbreviations

SAC	stent-assisted coiling
mRS	modified Rankin scale
DSA	digital subtraction angiography
AComA	anterior communicating artery
ACA	anterior cerebral artery
MCA	middle cerebral artery
ICA	internal carotid artery
BA	basilar artery
RROC	Raymond–Roy occlusion classification
